# Malignant inflammatory myofibroblastic tumor: a rare case presentation

**DOI:** 10.1093/jscr/rjac403

**Published:** 2022-09-19

**Authors:** Megan Spafford, Danley Lunn, Peter Graham

**Affiliations:** Department of General Surgery, University of Saskatchewan, Saskatoon, SK, Canada; Department of General Surgery, University of Saskatchewan, Saskatoon, SK, Canada; Department of General Surgery, University of Saskatchewan, Saskatoon, SK, Canada

## Abstract

Malignant inflammatory myofibroblastic tumors (IMT) are extremely rare, aggressive tumors with variable presentation. This is a case of a 29-year-old female presented with severe anemia and a large abdominal mass presumed to be a gastrointestinal stromal tumor (GIST). Severe anemia, leukocytosis and thrombocytosis accompanied the presentation. Final pathological assessment yielded a diagnosis of malignant IMT. Given the rarity of these tumors, no established diagnostic criteria exist aside from histological analysis of the tissue, which may result in delays or inappropriate treatment. As these tumors are aggressive in nature, a high index of suspicion is critical to improve outcomes. Further reports on the presentation, diagnosis and treatment of such rare tumors are important to develop clinical diagnostic guidelines to improve diagnosis and treatment and improve outcomes.

## INTRODUCTION

Inflammatory myofibroblastic tumors (IMT) are uncommon tumors first described in 1939 [1]. Patient presentation, treatment and clinical course are variable depending on the origin of the primary tumor [1]. They frequently present in a younger population and can be seen in any part of the body. Extra-pulmonary tumors have a 25% risk of local recurrence with less than 5% metastatic [2]. Diagnosis is made exclusively by pathological assessment of the tissue [3]. Here, we present a case of a malignant abdominal IMT in a young, adult female. Contributing to the literature on such a rare entity is crucial to help prevent diagnostic uncertainty and subsequent delays in treatment.

## CASE PRESENTATION

A 29-year-old female presented to hospital with abdominal pain. She had anemia (~110 g/L) and intermittent low-grade leukocytosis. She had been having persistent abdominal pain, intermittent nausea and drenching night sweats. She denied weight loss, fevers and reported no other notable symptoms. Her past medical history was unremarkable and past surgical history included two caesarean sections. She was not taking any medications and had no allergies. She was a non-smoker and social alcohol user. On examination, she was febrile with a temperature of 38.1°C, but all other vital signs were within normal limits. Her abdomen was tender to palpation, and there was a palpable mass in the epigastric region.

**Figure 1:**
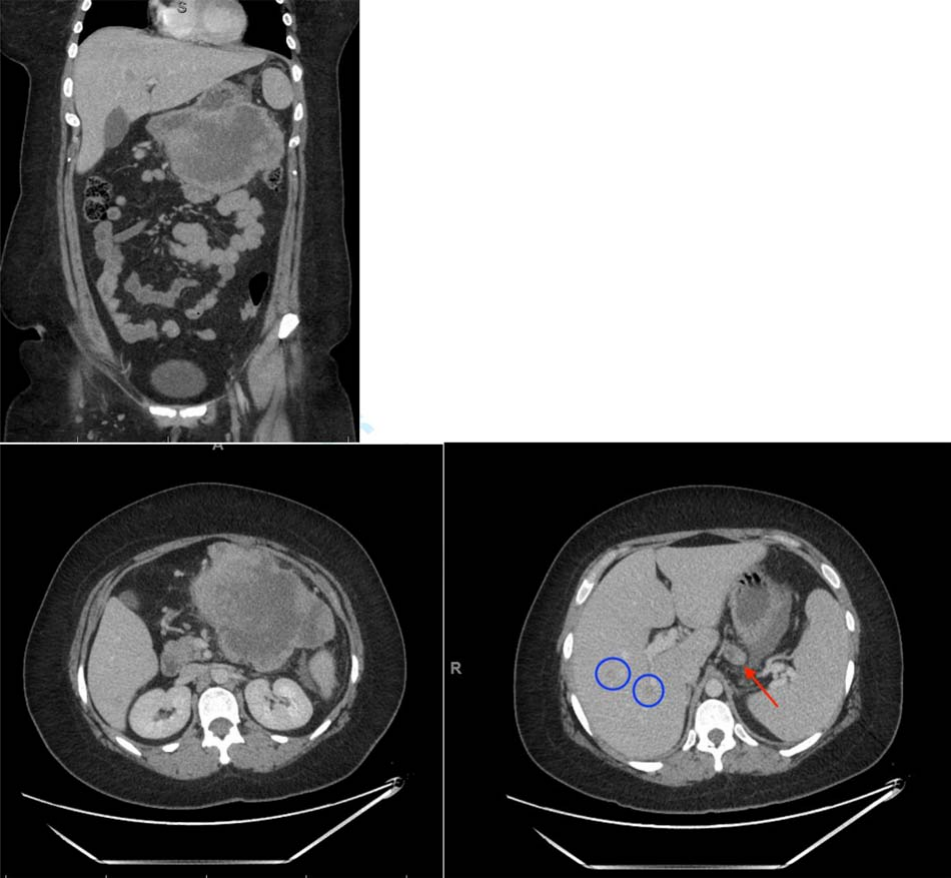
Initial CT scan showing large epigastric mass originating from the greater curvature of the stomach. Red arrow indicates large necrotic lymph node. Blue circles indicate liver metastases.

Investigations revealed an elevated WBC count of 35 × 10e9/L, a hemoglobin of 72 g/L and a CRP of 141 mg/L. A CT scan revealed a 15 × 11 cm multi-lobulated, heterogeneous, necrotic, exophytic mass arising from the stomach as well as multiple peritoneal deposits and five suspicious masses in the liver (Fig. 1). Upper endoscopy and liver biopsies were performed, and she was discharged home awaiting pathologic diagnosis. CT scan of her chest was negative for metastatic disease.

One month later, she re-presented with symptomatic anemia (hemoglobin of 56 g/L). Her leukocytosis had worsened, ranging from 38–44 × 10e9/L. She was urgently referred to a surgical oncologist for definitive management. Her biopsies reported a spindle cell neoplasm—CD117 and DOG-1 negative. However, given the anemia, the heterogeneous/ulcerated appearance on imaging, and the presence of liver metastases, a preliminary diagnosis of a GIST was made. After discussion at multidisciplinary tumor board rounds, and given her ongoing bleeding, the decision was made to proceed with upfront surgery. She underwent a subtotal gastrectomy, transverse colectomy, distal pancreatectomy, splenectomy, drainage of intraabdominal abscess and formation of an end colostomy. Intraoperative findings demonstrated infiltration of the mass into the transverse colon with perforation into the retroperitoneum. There were multiple omental nodules. Final pathology reported a negative gastric resection margin.

**Figure 2:**
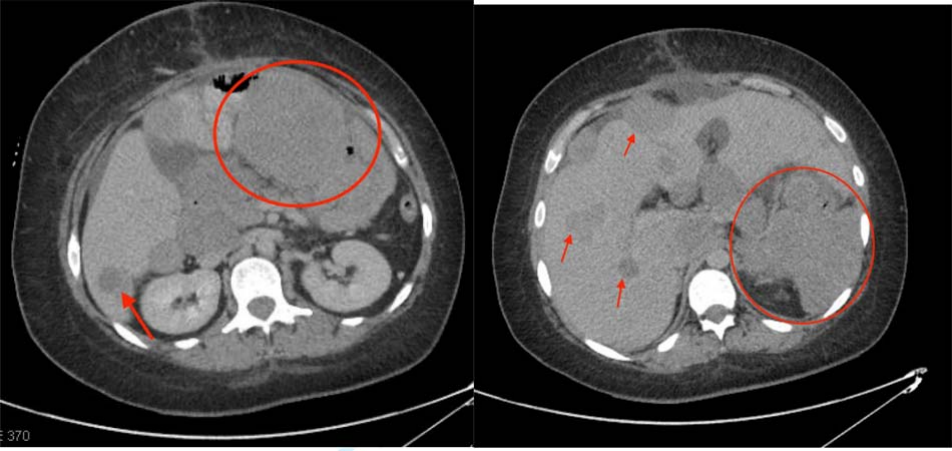
One-month post-operative follow-up CT scan showing progression of disease with large metastatic deposits (indicated by the red circles and arrows).

Postoperatively, her hemoglobin and leukocytosis improved (hemoglobin of 95 g/L and a WBC of 20 × 10e9/L); however, she developed a large pulmonary embolus and was placed on anticoagulation. A one-month follow-up CT scan showed progression of disease with a gastric mass measuring 6 cm, multiple enlarged mesenteric masses and enlargement of the hepatic metastases (Fig. 2). Final pathology analysis reported a malignant IMT with spindle cells mixed with patchy areas of inflammation and necrosis. The specimen was ALK and vimentin positive and pankeratin, C-kit, DOG-1, NTRK, smooth muscle actin, Melan A and HMB45 negative. The lesion had a high Ki67 proliferation index of 80–85%. She was started on Crizotinib (tyrosine kinase inhibitor), as it has shown to be effective in control of local recurrence in ALK positive IMTs [4].

In follow-up, she continued to have a gradually increasing WBC count reaching as high as 205 × 10e9/L and a persistent anemia despite ongoing transfusions. Another CT scan was performed showing significant progression of multiple large soft tissue masses in the abdomen as well as sclerotic osseous metastasis. Given the progression of disease, she was switched over from Crizotinib to Alectinib. Her disease continued to progress and 4 months following her surgery the patient passed away from her disease.

## DISCUSSION

IMT is a spindle cell tumor containing multiple inflammatory cells [5]. It has many aliases, most commonly known as ‘inflammatory pseudotumor’ and is therefore challenging to quantify an accurate incidence rate. It is most frequently reported in the lungs of children [2], but has been reported in abdominal organs, the retroperitoneum, heart, thyroid and kidney [3]. IMT’s tend to be locally aggressive, but the malignant version (presence of distant metastatic disease) is very rare, making up less than 5% [2]. There are no well-defined risk factors, and symptoms are often non-specific relating to the organ that the tumor is originating from [2].

IMTs are diagnosed histologically; however, histological characteristics do not provide any indication on prognosis [6]. Activation of ALK is present in 50% or more of patients with IMT [7], which provides guidance diagnosis as ALK positivity can help differentiate IMT from other histologically similar tumors [6]. In the few studies identifying ALK in IMT, the ALK positive IMTs tended to be in individuals with local recurrence, whereas the cases that were non-reactive for ALK were associated with death due to locally advanced or metastatic disease [6, 7]. Crizitonib, which has been shown to be effective in ALK positive non-small cell carcinoma, has also shown promising results in diffusely metastatic or unresectable ALK positive IMT, with a response rate averaging around 9 months [8]. However, the relationship between ALK positivity and response to treatment is not clear [6–8], which could explain the transient response our patient had to targeted therapy. Therefore, although targeted treatment remains an option, surgery is the treatment of choice.

Another unique quality that has been reported in cases of IMT is patients presenting with laboratory abnormalities such as anemia, leukocytosis and elevated platelet count [2, 9, 10]. These abnormalities generally resolve with complete surgical resection and appropriate treatment. Etiology is unknown but is believed to be secondary to inflammation and bone marrow reactivity; however, paraneoplastic syndrome cannot be ruled out and requires further research [10]. These specific lab abnormalities may be a key to earlier diagnosis for IMT, or at least provide a higher clinical suspicion.

Although there is a limited body of literature surrounding IMT, there are reports detailing a tendency for local recurrence, but with a small risk of distant metastasis [5]. As demonstrated in Gleason and Hornick’s paper, it is important to recognize that there are multiple, pathologically distinct tumors previously categorized under one broad umbrella of inflammatory pseudotumor. This raises the question as to just how many classes of tumors were buried within this category and therefore how different the treatment for each class will be. More data are needed to identify the similarities and differences of each to delineate the clinical significance between classes.

## CONCLUSION

IMT are locally aggressive tumors affecting predominantly young adults. The malignant variety including distant metastatic disease is rare. The cornerstone of treatment is a complete surgical resection but has evolved to include ALK targeted therapy. Rates of local recurrence are high (approximately 25%), and it remains a challenging disease to manage. Given the vague history in the current, albeit limited body of literature, increased reporting of these rare malignancies is needed to assist in diagnosing and treating it. As more case reports become available, we can begin to develop diagnostic, treatment, and follow-up guidelines to improve management and patient outcomes.

## CONSENT

Written informed consent was obtained from the husband of the patient for publication of this case report and accompanying images. A copy of the written consent is available for review by the Editor-in-Chief of this journal on request.

## CONFLICT OF INTEREST STATEMENT

No conflict of interest to disclose.

## FUNDING

This research did not receive any specific grant from funding agencies in the public, commercial or not-for-profit sectors.

## GUARANTOR

Provenance and peer review. Not commissioned, externally peer-reviewed.
